# Reformulation of Bologna Sausage by Total Pork Backfat Replacement with an Emulsion Gel Based on Olive, Walnut, and Chia Oils, and Stabilized with Chitosan

**DOI:** 10.3390/foods12183455

**Published:** 2023-09-16

**Authors:** Nicoleta Cîrstea (Lazăr), Violeta Nour, Alexandru Radu Corbu, Camelia Muntean, Georgiana Gabriela Codină

**Affiliations:** 1 Faculty of Food Science and Engineering, Dunărea de Jos University of Galati, Domnească Street 111, 800201 Galati, Romania; nl135@student.ugal.ro; 2 Department of Horticulture and Food Science, University of Craiova, A.I. Cuza Street 13, 200585 Craiova, Romania; alexandru.corbu@edu.ucv.ro (A.R.C.); camelia.muntean@edu.ucv.ro (C.M.); 3 Faculty of Food Engineering, Stefan cel Mare University of Suceava, 720229 Suceava, Romania; codina@fia.usv.ro

**Keywords:** Bologna sausage, emulsion gels, chitosan, reformulation, fatty acid composition, texture, technological properties

## Abstract

Bologna sausage, also called “la grassa”, is a very popular meat product despite its high fat content and lipidic profile raising serious negative health concerns. An emulsion gel containing olive, walnut, and chia oils, stabilized with soy protein isolate, transglutaminase, and chitosan, was used as total pork backfat replacer in Bologna sausage. The nutritional, textural, and technological properties were assessed and sensory analyses were conducted. Color, pH, and lipid oxidation were monitored during 18 days of cold storage (4 °C). A normal fat Bologna sausage was used as a control reference. A decrease in the n-6/n-3 ratio from 16.85 to 1.86 (by 9 times) was achieved in the reformulated product as compared with the control, while the PUFA/SFA ratio increased from 0.57 to 1.61. Color measurements indicated that the lightness and yellowness increased while redness slightly decreased in the reformulated product. The total substitution of pork backfat in Bologna sausage by the emulsion gel developed in the present study was realized without significantly affecting the technological properties, the oxidative stability and the overall acceptance by the consumers.

## 1. Introduction

Bologna sausage is a very popular emulsified sausage derived from the Italian mortadella, made of finely ground beef, pork, turkey, or chicken meat, stuffed into a casing, and then cooked or smoked. It is also called “la grassa” due to its high-fat content (ranging between 20% and 30%) dominated by saturated fats, and high energy value (around 300 kcal/100 g) [1], which can increase the risk of certain chronic diseases and contribute to weight gain. Bologna sausage constitutes an important product in the diet of many people due to its sensory appeal and affordable cost [2]. However, in recent years, consumers have increasingly focused on a healthier lifestyle and their needs regarding diets have changed. As a result, their interest in purchasing this type of product has decreased in order to comply with the recommendations for optimal fat intake and profile. To address this concern, the meat industry grapples with the challenge of researching and adopting different alternatives for producing healthier meat products, with a lower fat content and a better fatty acid profile [3,4,5,6]. One of the most current modalities to improve the fat profile in meat products is reformulation by reducing fat content and/or replacing the added animal fat (commonly pork backfat) with vegetable and/or marine oils, characterized by a lower content of saturated fatty acids (SFA) and a higher content of unsaturated fatty acids, both monounsaturated (MUFA) and polyunsaturated (PUFA) [7,8]. Animal fats possess intrinsic and unique features such as specific texture, mouth feel, juiciness, taste, and flavor, all of which positively contributes to the sensory acceptability of comminuted meat products. Some previous studies have already demonstrated that the direct substitution of animal fat with vegetable and/or marine oils in these products resulted in significant changes in textural, technological, yield, and sensory properties [9,10]. Moreover, the replacement could increase the susceptibility to oxidation of fat-rich meat products because of the higher unsaturation extent of the oils as compared with animal fats [11,12]. To solve these issues, meat scientists are lately investigating the incorporation of vegetable and marine oils in solid oil-structured emulsions such as oleogels or emulsion gels intended to be used as fat alternatives in reformulated meat products. The emulsion gels can be formulated by incorporating a cold gelling agent into a protein-stabilized emulsion containing the oil. The cold gelling agents are based on proteins, polysaccharides or their combinations, which can establish polymer interactions and create a gel-like network structure responsible for their functional properties [13,14,15]. These structures trapping oils have to be able to mime the textural properties of animal fats, to maintain the water and fat retention capacity of the meat product and to protect the incorporated lipids from oxidation [16,17,18].

Several studies have been conducted on the reformulation of finely comminuted cooked meat products such as Bologna [2,4,19] and frankfurter-type sausages [5,7,20,21] by using individual or combinations of healthy oils. In a recent study from our research team [12], a new emulsion gel formulated with a healthy oil mixture emulsified with soy protein isolate and using chitosan as a cold gelling agent proved to be an adequate substitute for pork backfat in pork patties. Although chitosan is a versatile natural polysaccharide with low-cost and highly efficient cross-linking ability, it was never used as a cold gelling agent in the emulsion gels incorporated as fat replacers in meat products. The incorporation of walnut oil in the emulsion gel along with the chia and olive oils is also an element of novelty of the present study and it had as a goal the improvement of the lipid profile and of the antioxidant protection of the meat product. The purpose of the present experiment was to assess the suitability of an emulsion gel containing olive, walnut, and chia oils stabilized with soy protein isolate, transglutaminase, and chitosan for use as pork backfat replacer in Bologna sausage in order to improve the lipid profile while preserving the sensory and technological properties of this popular meat product. Nutritional, textural and technological properties of the reformulated meat product were determined and sensory analyses were conducted. Physicochemical properties and lipid oxidation were monitored over storage for 18 days at 4 °C. The traditional Bologna sausage was used as a control reference.

## 2. Materials and Methods

### 2.1. Materials

Soy protein isolate (90% protein concentration) from Solae Belgium N.V. (Ieper, Belgium), microbial transglutaminase Activa WM, with an enzyme activity of 100 U/g, from Ajinomoto Europe Sales GmbH (Hamburg, Germany), and chitosan from BiOrigins (Fordingbridge, UK) were used to formulate the stabilizer system of the emulsion gel. The oil phase consisted of a mixture of 55% extra virgin olive oil (Monini, Spoleto, Italy), 25% extra virgin walnut oil (Hortus Verdi, Oradea, Romania), and 20% extra virgin chia oil (Huilerie de Lapalisse, Lapalisse, France).

According to suppliers’ information, the extra virgin olive oil contained 15.2% SFA, 75% MUFA, and 9.8% PUFA, the walnut oil contained 8.8.% SFA, 21.8% MUFA, and 60.8% PUFA, while for the extra virgin chia oil a lipid composition of 10.7% SFA, 9.5% MUFA, and 79.8% PUFA was specified.

### 2.2. Emulsion Gel Preparation

The emulsion gel was produced one day before the processing of Bologna sausage according to a slightly modified method of Pintado and Cofrades [18], tested by our research group in a previous study [12]. In brief, soy protein isolate (8%) and transglutaminase (1%) were dispersed under constant stirring for 2 min in water (43%) at room temperature in a planetary mixer (Rohnson R586, 700 W, Praha, Czech Republic), then adding chitosan (3%) as a cold gelling agent and continuing to homogenize for 3 min until complete mixing. The final blend was emulsified further for 3 min under stirring with continuous inclusion of the oil mixture (45%) described above. The resulting emulsion gel was then placed into plastic containers and stored under cold conditions (4 °C) for 20 h before sausage preparation. The color, pH, and thermal stability of the emulsion gel were determined immediately after obtaining as well as after 10 days of cold storage (4 °C).

### 2.3. Bologna Sausage Production

Fresh postrigor pork meat (mixture of *biceps femoris*, *semimembranosus*, *semitendinosus*, *gracilis*, and adductor muscles) and pork backfat from a local slaughterhouse were used for Bologna sausage preparation. Other ingredients and additives used were nitric salt, granulated garlic, and potato starch from Daz Activ (Bucharest, Romania), food coloring Propicolor, and additives and spices mixture for Bologna sausage from Helmut Grün (Bucharest, Romania) containing phosphates (E 451), lactose, sodium erythorbate (E 316), pepper, nutmeg, and cardamom. The control Bologna sausages were prepared according to the following formulation: pork meat (15 kg), pork back fat (4 kg), water (5 kg), starch (0.75 kg), nitric salt (0.40 kg g), additives and spices mixture for Bologna sausage (0.25 kg), granulated garlic (0.05 kg), and the food coloring Propicolor (0.02 kg).

**Figure 1:**
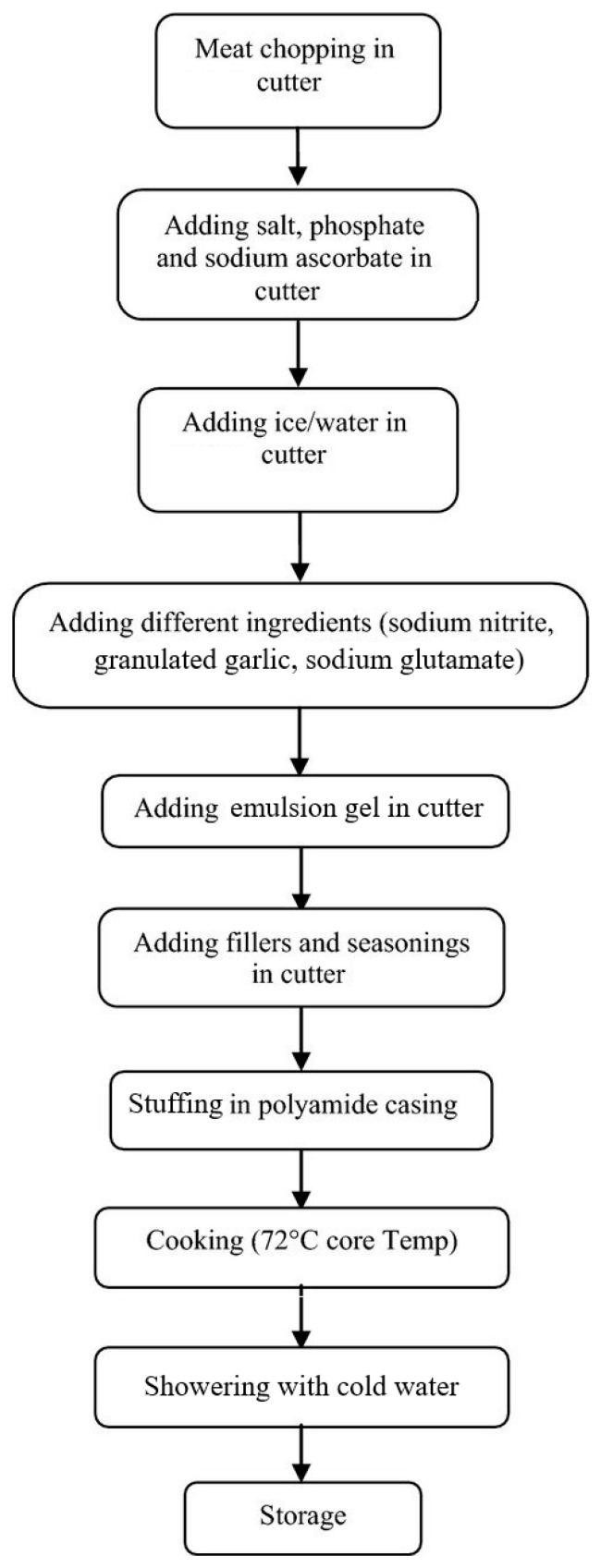
Flowchart of the process for the industrial production of Bologna sausages.

The sausages were stuffed into 100 mm diameter and 50 mm length artificial casings (Betex KDB 100/50 Salmon Pantone Pork, Betex-Pack, Belgium) using an HP-25 vacuum filler (Vemag Maschinenbau, Verden, Germany) and cooked in a smoking chamber Fessmann Turbomat 1800 RT (Fessmann, Winnenden, Germany) at 78 °C until the center of the sausages reached 72 °C (35 min). Then, the sausages were showered with cold water and stored at 4 °C for 18 days for further analysis. Figure 1 presents the flowchart of the process for the industrial production of Bologna sausages. Reformulated Bologna sausage (RBS) was produced based on the same formulation and technology as the control (CBS), except pork backfat was replaced with the emulsion gel. Batches of 25 kg were produced in triplicate for each formulation.

### 2.4. Proximate Analysis

The sausages were analyzed the day after processing for moisture, fat, protein, and ash contents following the AOAC official procedures [22], while carbohydrate content was estimated by difference. Each analysis was performed in triplicate. A drying oven (Memmert ULM500, Uden, The Netherlands) was used for the determination of the moisture content, a Soxhlet automatic extraction system (SER 148/3, Velp Scientific, Usmate, Italy) for the fat content, an automated nitrogen analyzer (UDK 149 Velp Scientific, Milan, Italy) for the protein content and a Caloris CL 1206 oven (Romania) for the ash content. Energy value was calculated based on 4 kcal/g for carbohydrate and protein content and 9 kcal/g for fat content.

### 2.5. Fatty Acid Profile and Nutritional Indices

The fatty acid composition was analyzed in triplicate by fatty acid methyl ester (FAME) gas chromatography using a Perkin-Elmer gas chromatograph model Clarus 500 (Shelton, MA, USA). The fatty acids from the lipid extracts of the sausage samples were transesterificated at 80 °C in methanol containing 3% concentrated sulfuric acid to be converted to their methyl esters (FAMEs). The separation of FAMEs was performed on a DB-23 GC capillary column (60 m × 0.25 mm id × 0.25 µm film thickness) from Agilent J & W GC Columns (Santa Clara, CA, USA). The initial column temperature was 180 °C, the ramp rate was 5 °C/min, and the final temperature was 220 °C. Hydrogen was used as a carrier gas at a flow rate of 35 cm/s. FAMEs were detected with a flame ionization detector (FID) and identified by comparing retention times with those of the individual standards from Sigma-Aldrich Chemical Co. (Saint Louis, MO, USA). The results were expressed as grams of fatty acid per 100 g of total fatty acids. The results were used to calculate the total sum of saturated (Σ SFA), monounsaturated (Σ MUFA), polyunsaturated (Σ PUFA), n-3 polyunsaturated (Σ n-3 PUFAs), and n-6 polyunsaturated (Σ n-6 PUFAs) fatty acids and the n-6/n-3 ratio. The atherogenic (AI) and thrombogenic (TI) indexes were calculated from the fatty acid profile according to Ulbricht and Southgate [23]:

AI (Atherogenic Index) = (C12:0 + 4 × C14:0 + C16:0)/(Σ MUFA + Σ PUFA)

TI (Thrombogenic Index) = (C14:0 + C16:0 + C18:0)/(0.5 × Σ MUFA + 0.5 × Σ PUFA n-6 + 3 × Σ PUFA n-3 + PUFA n-3/PUFA n-6)

The hypocholesterolemic and hypercholesterolemic fatty acids ratio (h/H) was calculated according to Santos-Silva et al. [24]:

h/H (hypocholesterolemic/Hypercholesterolemic index) = (C18:1 n-9 + C18:2 n-6 + C20:4 n-6 + C18:3 n-3 + C20:5 n-3 + C22:5 n-3 + C22:n-3)/(C14:0 + C16:0)

### 2.6. pH Measurement

The pH was determined in the sausage homogenate by using a Hanna model HI255 pH-meter (Hanna Instruments, Padova, Italy) [9]. The homogenates were prepared in water in a ratio of 1:10 (*w*/*v*) at room temperature. Three determinations were performed for each formulation.

### 2.7. Thermal Stability of Emulsion Gels

The thermal stability of emulsion gels, as the total fluid release, was evaluated in triplicate following a slightly modified method of Jiménez-Colmenero et al. [20]. Samples of emulsion gel (about 25 g) were stuffed and hermetically sealed in plastic tubes and heated in a water bath at 70 °C for 30 min. The tubes were centrifuged for 15 min at 2500× *g* in a Hermle Z300 centrifuge (Hermle Labortechnik, Wehingen, Germany) and left standing upside down for 50 min to leak out the separated exudate (fat and water). The remanent emulsion was weighed and the total fluid release was expressed as g/100 g of initial sample weight.

### 2.8. Thermal Processing Loss and Moisture Retention

The cooking loss, occurring after heat processing and chilling overnight at 2 °C, was calculated by the weight difference between raw and cooked sausages and expressed as a percentage of the raw weight [9]:

Cooking loss (%) = [(weight_raw_ − weight_cooked_)/weight_raw_] × 100

The moisture retention, representing the quantity of moisture retained in the cooked product per 100 g of raw sample, was calculated as follows [25]:

Moisture retention (%) = [(100 − cooking loss (%)) × moisture_cooked_]/100

### 2.9. Color Measurement

The color of the sausages was assessed the day after processing and after 4, 8, 12 and 18 days of storage from six fresh-cut sections at three different points of each section as described by Cîrstea et al. [9]. The color coordinates L* (lightness), a* (redness), and b* (yellowness) were measured on a PCECSM1 colorimeter (PCE Instruments, Southampton, UK) with spectral reflectance, operating in the CIEL*a*b* system, calibrated against a white standard.

### 2.10. Texture Profile Analysis

To analyze the texture of the sausages, a 10 kg load cell was used on a TVT-6700 texture analyzer from Perten Instruments (Hagersten, Sweden). The textural features of the samples were characterized according to Saracila et al. [26] using the following determinants: hardness, springiness, resilience, gumminess and cohesion. For this analysis, sample slices were cut from the center of the product, whose height was 1.5 cm. Two compression cycles were used on the sectioned slices with a maximum of 50% of their initial weight. This determination required a 20 mm cylindrical probe and a trigger force of 20 g at a speed of 1.5 mm/s, with 10 s between compressions used as recovery time. For each sample, eight determinations were made. The Warner–Bratzler shear test was carried out to determine the shear force (N) as an indicator of firmness, with each sample being assessed three times. The sample used was 100 mm in height and we compressed it by 40 mm at a 5 mm starting point from the sample. This compression was carried out at a 1.5 mm/s initial speed and test speed, with a trigger force of 40 g and 10 mm/s retracting speed at a data rate of 200 pps. The highest level of shear force was used to the strongest resistance point of the sample to shearing, which is at the top of the curve.

### 2.11. Lipid Oxidation

The oxidative stability of sausages was assessed by the evolution of thiobarbituric acid-reactive substances (TBARS) during 18 days of refrigerated storage. TBARS values were determined according to Witte et al. [27] and Ben Hsouna et al. [28] as follows: sausage samples (5 g) were vortexed in 12.5 mL of 20% trichloroacetic acid, then transferred to a 25 mL volumetric flask and diluted up to the volume with cold distilled water. After centrifugation at 2500× *g* for 5 min, 5 mL of supernatant was collected and mixed with 5 mL of 0.02 M 2-thiobarbituric acid. Finally, this mixture was heated at 100 °C during 35 min and, after cooling to room temperature, the absorbance was read at 532 nm on a Varian Cary 50-UV spectrophotometer (Varian Co., Palo Alto, CA, USA). TBARS values were expressed as mg malonaldehyde (MDA)/kg of sausage based on a standard curve prepared from 1,1,3,3-tetraethoxypropane.

### 2.12. Sensory Analysis

Control and reformulated Bologna sausages were evaluated the day after processing in terms of color, taste, flavor, texture and overall acceptability. A 9-point hedonic scale ranging from 1 denoting “dislike extremely” to 9 denoting “like extremely” was used. The panel consisted of 24 members selected among staff and master students of the Food Science Department of the University of Craiova (Craiova, Romania). The samples were offered to the panelists in a randomized order after removal from cold storage. Triplicate evaluations were made for each sample and the average score was calculated for each attribute.

### 2.13. Statistical Analysis

Data statistical analysis was carried out using the Statgraphics Centurion XVI software (StatPoint Technologies, Warrenton, VA, USA). The statistical significance of the effect of sausage formulation on proximate and fatty acid composition, texture, technological properties, and sensory attributes was evaluated by applying the one-way ANOVA (analysis of variance) with the least significant difference (LSD) test at a 5% level of probability. Additionally, two-way ANOVA accompanied by LSD multiple comparison test (*p* < 0.05) was performed to investigate the effect of formulation and storage period on pH values, color parameters, and TBARS values.

## 3. Results

### 3.1. Color, pH, and Thermal Stability of the Emulsion Gel

The color, pH, and thermal stability of the emulsion gel were assessed immediately after processing and after 10 days of cold storage (4 °C) (Table 1).

The color of the emulsion gel as well as its evolution during storage are of interest considering that they could determine color changes in the reformulated meat products. The emulsion gel obtained and used in this study had a yellowish-cream color (b* = 20.63) which influenced to some extent the color of the Bologna sausage (Figure S1, available in the Supplementary Materials). L* values significantly increased while a* and b* values significantly decreased (*p* < 0.05) after 10 days of storage at 4 °C. These changes in the color parameters assert a perceptible loss of color intensity during storage, mainly caused by the decrease of yellowness (b* values).

A similar evolution of the color CIELab values during storage was previously reported by Alejandre et al. [14] in a gelled emulsion incorporating carrageenan and algae oil and formulated as an animal fat replacer in different meat products. They attributed the loss of yellowness to the isomerization and degradation of carotenoids from vegetal oils caused by drying and lipid oxidation.

The pH of the emulsion gel was high as compared with the other emulsion gels formulated in previous studies and this was mainly due to the incorporation of chitosan. Jiménez-Colmenero et al. [20] reported the highest pH (7.71) in the emulsion gel stabilized with sodium caseinate, while the rest of the emulsions formulated by them using soy protein isolate, microbial transglutaminase, and meat protein had pH values ranging between 7.4–7.5. In the present study, the emulsion gel presented a solid-like structure with good stability and no syneresis or exudate release during processing or storage. Similar good phase separation stabilities have been previously reported in gelled emulsions stabilized with gelatin, sodium alginate, or their mixture [29] in emulsion gels containing chia flour and cold gelling agents (transglutaminase, alginate, or gelatin) [15], or in polysaccharide gel matrices prepared using mixed biopolymer systems of alginate with inulin or dextrin [16].

The use of emulsion gels in thermal-processed meat products makes their thermal stability a technological characteristic of major interest. The emulsion gel formulated in the present study showed excellent thermal stability both initially and after 10 days of storage, as heating produced low levels of fluid release (1.01% after processing and 1.37% after 10 days of storage).

The emulsion gels formulated in some previous studies using sodium caseinate, soy protein isolate, meat protein, and microbial transglutaminase exhibited also good thermal stabilities [20,30]. The incorporation of 1% transglutaminase contributed to the stability and the emulsifying properties of the gel matrix and of the final meat product as it has been shown that transglutaminase determines crosslinking of soy proteins as well as soluble myofibrillar proteins and facilitates the interactions between meat and soy proteins [20].

### 3.2. Proximate Composition and Energy Values

The proximate composition of the control and reformulated Bologna sausages is shown in Table 2. The replacement of pork backfat with the emulsion gel slightly affected the proximate composition and energy value of Bologna sausage.

The reformulation of Bologna sausage determined a slight increment in the protein content and a small decrease in the moisture content that reached statistical (*p* < 0.05) significance. Franco et al. [5] also reported slight reductions in the moisture content and slight increases in the protein content after the reformulation of frankfurter sausages by replacing 25% and 50% of pork backfat with a linseed oleogel while Jiménez-Colmenero et al. [20] found no differences (*p* < 0.05) in the moisture content (ranging from 60.6 to 62.3%) yet a slightly higher protein content in reformulated frankfurters made by the replacement of pork backfat with oil-in-water emulsions formulated using sodium caseinate, soy protein isolate, meat protein, and transglutaminase, as compared with control frankfurters. The small differences in protein content between control and reformulated sausages could be justified by the fact that the emulsion gel was formulated with 8% soy protein isolate and 1% transglutaminase while the pork backfat contained 8.58% protein. Although a slight decrease was observed, no statistical differences were found between control and reformulated sausages when it comes to fat content (*p* < 0.05), explained since the oil mixture represented 45% of its formulation. In good agreement with these findings, some previous works found that the reformulation of frankfurter sausages [5] and dry-fermented sausages [6] with oleogels based on beeswax-linseed oil did not significantly modify the fat content. Pérez-Álvarez et al. [21] found also no statistical differences (*p* > 0.05) in the fat content of control and reformulated Frankfurt-type sausages with 50% and 75% fat substitution by chia-mucilage egg white-based oleogels.

However, the fat content was very low both in control and reformulated Bologna sausages from the present study as compared with those previously reported. Franco et al. [5] found values of the fat content between 18.35% and 20.03% in control and reformulated frankfurter sausages, Barbut et al. [9] reported 20.8% fat content in breakfast sausages while Domínguez et al. [3] found 14.41% in frankfurter type sausage made with microencapsulated fish oil. However, fat contents of around 10.3% have been reported for n-3 PUFA-enriched frankfurters elaborated with healthier oils stabilized in a konjac matrix as fat replacers [13].

In the present study, energy values of 191.98 kcal/100 g and 198.77 kcal/100 g were found in control and reformulated Bologna sausages (based on 9 kcal/g for fat and 4 kcal/g for protein), of which 58.8% and 56.1% were supplied by fat, respectively. Previously, Jiménez-Colmenero et al. [20] reported a caloric content of 225–245 kal/100 g in control and reformulated frankfurters of which around 70% was delivered by fat.

The energy provided by the fat decreased slightly but not significantly (*p* < 0.05) in reformulated sausages as compared with the controls while the energy value had a small increase. As previously found, ash content was not significantly affected by the substitution of pork backfat with emulsion gels [10,11].

### 3.3. Fatty Acids Profile

The influence of the total replacement of pork backfat with the emulsion gel on the fatty acid profile and nutritional indices of Bologna sausage is shown in Table 3.

As shown in previous studies where vegetable and marine oils were incorporated into cooked meat products to replace animal fat [6,7,9,10,18,21], fatty acid content was impacted depending on the fatty acid profile of the oils and the level of replacement.

The most predominant fatty acids in the control sausage were monounsaturated fatty acids (MUFA), followed by saturated fatty acids (SFA) and polyunsaturated fatty acids (PUFA). In the control product, oleic acid ranks first in content followed by palmitic, linoleic, and stearic acids. Oleic acid remained the most predominant fatty acid in the reformulated product, but it was followed by linoleic, palmitic and α-linolenic acids. The palmitic acid content decreased significantly (*p* < 0.05) from 21.49 to 15.23 g/100 g of total fatty acids in the control and reformulated sausage, respectively. The stearic acid content showed a significant reduction from 9.31 to 5.74 g/100 g of total fatty acids. The SFA content was reduced by around 32.5% while the PUFA content increased 1.88 times in the reformulated Bologna sausage as compared with the control product. A much smaller SFA reduction (in the range of 3.4–7.99%) was accomplished as a result of the replacement of pork backfat with a linseed oleogel (at 50% level) in frankfurter sausages [5] or after the replacement of pork backfat with olive and sunflower oil hydrogel emulsions in goat meat burgers (15.98% and 19.15%, respectively) [10]. The MUFA content of normal fat sausage (48.25%) diminished to 41.60% in the reformulated product. Similar decreases in the MUFA content have been previously reported as a result of the partial or total substitution of pork backfat with oleogels in reformulated meat products [31].

Of the PUFAs, PUFA n-6 content increased 1.3 times but, by far, the most spectacular increase was achieved for the PUFA n-3 content which multiplied 12 times in the reformulated product, mainly as a consequence of the increase in the α-linolenic acid content (C18:3n-3) from 1.06 to 12.28 g/100 g. As a result, a strong decrease in the n-6/n-3 ratio, from 16.85 to 1.86 (by 9 times) was accomplished in the reformulated product as compared with the control, while the PUFA/SFA ratio raised from 0.57 to 1.61.

In good agreement with our results, Franco et al. [5] also reported a decrease in the n-6/n-3 ratio from 14.92 to 1.61 as a result of the replacement of 50% of pork backfat by a linseed oleogel, and the increase of the PUFA/SFA ratio from 0.47 to 0.78. Moreover, Jiménez-Colmenero et al. [32] reported a notable decrease in the n-6/n-3 ratio (11.25 versus 1.19) in dry fermented sausage by replacing the pork backfat with a healthier oil combination (olive, linseed and fish oils) stabilized in a konjac matrix, while Delgado-Pando et al. [7] found a decrease in the n-6/n-3 PUFA ratio from 9.27 to 0.47 and an increase in the PUFA/SFA ratio from 0.27 to 1.7 after the substitution of pork backfat with healthier oil-in-water emulsions in low-fat frankfurters.

The increase of the n-3 PUFA content in the reformulated sausage reflected the beneficial fatty acid composition of chia and walnut oils that account for 45% of the oil mixture used in this study. Chia oil has a high content of polyunsaturated fatty acids (>80%), of which around 60% is α-linolenic acid (18:3n-3) [33] while walnut oil is known also to possess one of the highest content of PUFAs (up to 78% of the total fatty acid content), with a noticeable amount of α-linolenic acid (8–15.5%) [34,35].

The diminution of the n-6/n-3 ratio is important for developing healthier meat products as the epidemiological studies demonstrated that the eicosanoids derived from n-3 PUFAs are anti-inflammatory while a high intake of n-6 PUFAs creates pro-inflammatory effects, thus increasing the risk of developing several diet-related chronic diseases [36].

The n-6/n-3 ratio was in the range considered healthy (1:1 to 2:1) in the reformulated Bologna sausage, which is in agreement with the dietary recommendations [37]. As a consequence, the replacement of pork backfat with the emulsion gel formulated in the present study considerably improved the healthiness of Bologna sausage by decreasing both the atherogenic index (0.41 in control vs. 0.24 in the reformulated sausage) and thrombogenic index (0.89 in control vs. 0.31 in the reformulated product), and by increasing the h/H ratio from 2.69 in control to 4.63 in the reformulated product.

Taking into account the results obtained concerning the reduction of the SFA content, the reformulated Bologna sausage can be claimed as having “reduced saturated fat” according to European Regulations [38]. In addition, the reformulated sausage can be also claimed as having “high unsaturated fat” and “high content of omega-3”, as at least 70% of the fatty acids present in the product derive from unsaturated fat and the product contains at least 0.6 g alpha-linolenic acid per 100 g as listed in the Corrigendum to Regulation (EC) No 1924/2006 [39] and Commission Regulation No 432/2012 [40].

### 3.4. Color Properties and pH

Color is one of the main parameters in the reformulation of meat products as the changes in color may influence consumer acceptability [41]. The reformulation had some effects on the color parameters of the Bologna sausages (Table 4).

The reformulated sausages were significantly lighter (higher L* values) (*p* < 0.05) and displayed significantly higher b* values (*p* < 0.05) than the control product containing pork backfat. However, the redness (a* values) only slightly decreased upon the total substitution of pork backfat with the emulsion gel. Martins et al. [42] attributed the lightness increase in reformulated products to the glassy appearance of the oleogels used for pork backfat substitution. The increase in yellowness as a result of the reformulation may be attributed to the yellowish-cream color of the emulsion gel, determined in turn by the color of the soy protein isolate and of the oil mixture. Other studies reported an increase in lightness and yellowness as a result of the fat substitution with emulsion gels based on vegetal oils [3,5,6,10,43]. However, yellowness was not strongly affected in the present study, the increment of b* values was only 8.9%, much lower than that reported by Franco et al. [6] (between 25.75% and 62.45%) in fermented sausages reformulated by partial replacement of pork backfat with linseed oil oleogels. The reduction in redness after the substitution of animal fat with emulsion gels in meat products has been also previously reported [5,44]. Figure S2 (available in the Supplementary Materials) presents the appearance of control and reformulated Bologna sausages immediately after processing.

pH values significantly (*p* < 0.05) increased as a result of reformulation due to the higher pH (7.61 ± 0.07) of the emulsion gel as compared with the pork backfat used in the control formulation (6.31 ± 0.06). A higher pH is expected to have a positive impact on the water-holding capacity in meat products due to the direct action of pH on the myofibrillar proteins’ net charge and filament spacing [45,46]. The pH raised during storage both in control and reformulated sausages as a result of the alkaline nitrogenous compounds produced through increased protein decomposition under the action of endogenous enzymes and bacteria.

### 3.5. Technological Properties

The results of cooking loss and moisture retention of sausages are shown in Table 5. Only a slight increase in cooking loss was recorded when manufacturing the new product as compared to the control product, accompanied by a reduction in moisture retention. However, the values indicated good thermal behavior of the emulsion gel in the meat product and good fat and water-binding properties. These effects may be related to the good thermal stability of the emulsion gel (Table 1), as well as to the higher protein content in the reformulated product (Table 2).

Other previous studies have also found that the replacement of pork backfat with emulsion gels had no or little effect on the cooking loss or processing yield of different sausages [9,20].

### 3.6. Texture Analysis

The changes in the textural properties represent one of the main challenges in the reformulation of sausages. The substitution of pork backfat with emulsion gel resulted in significantly firmer sausages (Table 6). In good agreement with these findings, some previous studies also reported that partial or total substitution of animal fat with various structured vegetable oils in finely comminuted meat products resulted in firmer products [9,47].

According to Youssef and Barbut [48], the increase in hardness may be attributed to the larger surface area of the protein membrane or interfacial protein film formed around the smaller vegetable oil globules dispersed in the meat batter that could increase the product’s resistance to compression. In addition, the increase in hardness may be attributed to the higher moisture and protein content of the reformulated products as a higher protein content acting to form the meat matrix is generally associated with harder structures [11]. Other studies also reported strong negative correlations between moisture content and hardness in dry fermented meat products [6,18,49].

All the other textural properties except cohesiveness were also significantly higher (*p* < 0.05) for the reformulated sausage as compared to the control (Table 6), demonstrating that the emulsion gel has a firm structure, with flexible characteristics, which mimics the textural characteristics of pork backfat, and consequently, may be used to replace the pork backfat fat in the Bologna sausage. Similar increases in textural characteristics have been reported in previous studies as a result of the partial or total replacement of pork backfat with emulsion gels in different sausages [5,9]. However, the differences in textural attributes found in the present study were relatively slight and might not even be detectable by most consumers. Cohesiveness was not affected by the reformulation, demonstrating the same binding activity provided by the emulsion gel as the pork backfat. The addition of transglutaminase contributed to the increase in hardness and chewiness of the reformulated product as a consequence of its role in stabilizing the emulsions made with soy protein and in promoting the formation of a much more stable gel matrix by crosslinking soy and soluble myofibrillar proteins [50].

### 3.7. Lipid Oxidation

The high polyunsaturation level of the oil mixture incorporated in the emulsion gel in the present study could be an important factor in making reformulated Bologna sausages highly susceptible to oxidation during storage, with major effects on their sensory characteristics and production of toxic compounds [51]. The fat content in control and reformulated products did not show statistical differences (*p* > 0.05) (Table 2) but the processing conditions, especially the fine grinding and cooking, contribute to the acceleration of the oxidation processes as a result of the enhanced access of oxygen to the substrate and exposure to high temperatures [7].

**Figure 2:**
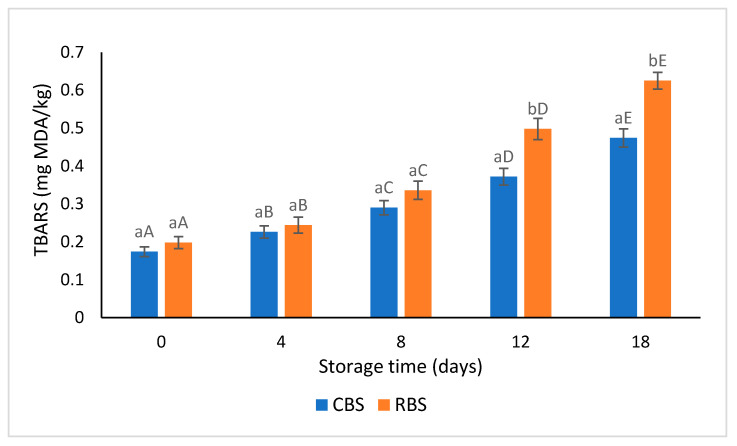
TBARS (mg MDA/kg) values of control and reformulated Bologna sausages at 0, 4, 8, 12, and 18 days of storage at 4 °C. Different lowercase letters indicate significant differences between sausage formulations (*p* < 0.05) for the same storage period, while different uppercase letters are indicative of significant differences between sampling times for the same sausage formulation (*p* < 0.05); CBS—control Bologna sausages; RBS—reformulated Bologna sausages.

The evolution of TBARS values in control and reformulated Bologna sausages during 18 days of cold storage is shown in Figure 2. Higher TBARS values were found in the reformulated product as compared with the control throughout the entire storage period, indicating that the replacement of pork backfat by the emulsion gel incorporating vegetable oils with a high unsaturation degree in sausage formulation promoted a greater extent of lipid oxidation. In addition, the incorporation of microbial transglutaminase in the stabilization system of the emulsion gel could have also contributed to the intensification of lipid oxidation [7], probably due to interference of the enzyme in the antioxidative mechanisms of proteins, thus reducing their ability to inhibit lipid oxidation. However, the differences between the TBARS values of the two samples became significant (*p* < 0.05) only after 12 days of refrigerated storage.

As expected, TBARS values significantly (*p* < 0.05) increased during storage, ranging from 0.174 mg MDA/kg to 0.625 mg MDA/kg. These values are similar to those reported by other studies in Frankfurt-type cooked sausages [7,21]. However, the TBARS values in all samples were lower than those reported to be the minimum detectable level (1.36 mg MDA/kg) for unpleasant oxidized flavors in processed meat products [52], indicating low lipid oxidation both in control and reformulated Bologna sausages. The good lipidic stability of the reformulated product was probably provided by the capacity of the emulsion gel to trap the oil mixture, acting as a barrier to oxygen and protecting the lipid fraction in the meat system [53], as well as by the oxidative stability of the soy protein isolate [54]. The natural antioxidant compounds occurring in the olive-walnut-chia oil mixture could also contributed to the decrease in lipid oxidation in the reformulated product. Virgin olive oil is well known for its high content of tocopherols and phenolic compounds that are strong antioxidants and radical scavengers [55,56]. Walnut oil also contains tocopherols, phytosterols, squalene, and polyphenols that provide good antioxidant capacities [57]. Moreover, chitosan could contribute to the inhibition of lipid oxidation owing to its redox-regulatory activity [58].

### 3.8. Sensory Analysis

Consumers are increasingly interested in functional meat products, but they are not willing to make sensory compromises for healthier reformulated products [59]. In addition to the technological and textural aspects, replacing pork backfat in meat products is a sensorial challenge as the pork backfat plays a significant role in the juiciness, tenderness, and flavor intensity of sausages [5]. Table 7 shows the mean scores for various sensory attributes and the overall acceptability of control and reformulated Bologna sausages. Except for the flavor, the scores were higher for the control product, yet the differences were not significant (*p* < 0.05).

The control product received a higher score for texture, which may be attributed to its higher juiciness being in good correlation with the higher moisture retention, and lower hardness, as presented previously. The reformulated product also received a lower score for color, likely due to the slightly yellowish tint of the reformulated Bologna sausage, as it is well known that in this type of product a higher yellowness suggests a lower acceptability [5]. However, neither the scores for texture nor those for color differed significantly (*p* > 0.05) between the control and reformulated sausages.

The reformulated product was rated better in terms of flavor, probably due to the aromas imparted by the olive and walnut oils, though the differences were barely noticeable. There were no significant differences (*p* > 0.05) between control and reformulated sausages regarding the scores for overall acceptability, the reformulated product being very well accepted by the panelists. These findings agree with other studies reporting that the reformulated frankfurters awarded similar scores to controls in terms of color, flavor, taste, and overall acceptability [6,38,60].

## 4. Conclusions

An emulsion gel with good thermal stability was successfully structured using soy protein isolate, transglutaminase, and chitosan as cold gelling agents into a solid-like structure containing an oil mixture made of extra virgin olive, walnut, and chia oils. The replacement of pork backfat with the emulsion gel considerably improved the Bologna sausage lipid-healthiness by reducing the saturated fatty acids content, n-6/n-3 ratio, AI, and TI due to the healthy fatty acid profile of the oil mixture incorporated in the reformulated product. The reformulated Bologna sausage can be claimed as “high unsaturated fat” and “high content of omega-3”, according to European regulations. Firmness, springiness, gumminess, resilience, and shear force were higher in the reformulated Bologna sausage as compared with the control, yet the differences were small and scarcely detectable by consumers. Lightness and yellowness increased in the reformulated product while redness slightly decreased, but the color of the reformulated sausage was not strongly affected by the replacement as indicated by the results of the sensory analysis. The reformulated product developed in the present study could be a healthier alternative to the traditional Bologna sausage since the technological properties, the oxidative stability and the overall acceptance by the consumer were only slightly affected by the reformulation.

## Figures and Tables

**Table 1:** Color parameters (L*—lightness, a*—redness, and b*—yellowness), pH, and total fluid release of emulsion gel at 0 and 10 days of storage at 4 °C *.

Parameter	Storage Time (Days)	
	0	10
L*	70.59 ± 3.61 ^a^	79.43 ± 1.77 ^b^
a*	1.53 ± 0.20 ^b^	0.17 ± 0.08 ^a^
b*	20.63 ± 0.95 ^b^	16.90 ± 0.23 ^a^
pH	7.61 ± 0.07 ^a^	8.21 ± 0.06 ^b^
Total fluid release (%)	1.01 ± 0.05 ^a^	1.37 ± 0.08 ^b^

* Different superscript letters indicate significant differences between sampling times (*p* < 0.05).

**Table 2:** Proximate composition and energy values of control and reformulated Bologna sausages *.

	CBS	RBS
Moisture (%)	68.31 ± 0.87 ^b^	66.47 ± 0.28 ^a^
Protein (%)	11.58 ± 0.23 ^a^	12.28 ± 0.39 ^b^
Fat (%)	12.41 ± 0.23 ^a^	12.26 ± 0.26 ^a^
Ash (%)	2.31 ± 0.10 ^a^	2.24 ± 0.13 ^a^
Energy value (kcal/100 g)	191.98 ± 1.88 ^a^	198.77 ± 2.08 ^b^
Energy from fat (kcal/100 g)	112.93 ± 2.09 ^a^	111.56 ± 1.68 ^a^

* Different superscript letters in the same raw indicate significant differences between sausage formulations (*p* < 0.05). CBS—control Bologna sausages; RBS—reformulated Bologna sausages.

**Table 3:** Fatty acid profile (expressed as g/100 g of total fatty acids) and nutritional indices of control and reformulated pork patties *.

Fatty Acids	CBS	RBS
Caprylic (C8:0)	0.16 ± 0.01 ^a^	0.08 ± 0.01 ^a^
Capric (C10:0)	0.17 ± 0.01 ^b^	0.14 ± 0.01 ^a^
Myristic (C14:0)	1.45 ± 0.07 ^b^	0.75 ± 0.04 ^a^
Pentadecenoic (C15:1n-13)	0.10 ± 0.00 ^a^	0.10 ± 0.01 ^a^
Palmitic (C16:0)	21.49 ± 0.96 ^b^	15.23 ± 0.48 ^a^
Palmitoleic (C16:1n-7)	3.64 ± 0.15 ^b^	1.77 ± 0.18 ^a^
Heptadecanoic (C17:0)	0.09 ± 0.01 ^b^	0.19 ± 0.01 ^a^
Heptadecenoic (C17:1n-7)	0.21 ± 0.02 ^b^	0.14 ± 0.01 ^a^
Stearic (C18:0)	9.31 ± 0.34 ^b^	5.74 ± 0.26 ^a^
Oleic (C18:1n-9)	44.30 ± 1.31 ^b^	39.59 ± 1.36 ^a^
Linoleic (C18:2n-6)	15.92 ± 0.56 ^a^	21.88 ± 0.62 ^b^
α Linolenic (C18:3n-3)	1.06 ± 0.05 ^a^	12.28 ± 0.34 ^b^
Octadecatetraenoic (C18:4n-3)	0.00 ± 0.00 ^a^	0.17 ± 0.01 ^b^
Eicosadienoic (C20:2n-6)	0.46 ± 0.03 ^b^	0.30 ± 0.02 ^a^
Arachidonic (C20:4n-6)	0.41 ± 0.02 ^b^	0.22 ± 0.01 ^a^
Tricosanoic (C23:0)	0.12 ± 0.01 ^b^	0.00 ± 0.00 ^a^
Docosadienoic (C22:2n-6)	1.07 ± 0.04 ^b^	0.76 ± 0.04 ^a^
Other fatty acids	0.04 ± 0.01 ^a^	0.66 ± 0.03 ^b^
Nutritional indices		
Σ SFA	32.79 ± 1.36 ^b^	22.13 ± 0.95 ^a^
Σ MUFA	48.25 ± 1.38 ^b^	41.60 ± 1.05 ^a^
Σ PUFA	18.92 ± 0.55 ^a^	35.61 ± 1.25 ^b^
Σ PUFA n-6	17.86 ± 0.49 ^a^	23.16 ± 0.86 ^b^
Σ PUFA n-3	1.06 ± 0.05 ^a^	12.45 ± 0.49 ^a^
n-6/n-3	16.85	1.86
AI	0.41	0.24
TI	0.89	0.31
h/H	2.69	4.63

* Different superscript letters in the same raw indicate significant differences between sausage formulations (*p* < 0.05). CBS—control Bologna sausages; RBS—reformulated Bologna sausages.

**Table 4:** Color parameters (L*—lightness, a*—redness, and b*—yellowness) and pH of control and reformulated Bologna sausages at 0, 4, 8, 12 and 18 days of storage at 4 °C *.

Parameter	Samples	Storage Time (Days)				
		0	4	8	12	18
L*	CBS	76.62 ± 0.98 ^a,B^	74.98 ± 1.67 ^a,A^	75.67 ± 0.63 ^a,A,B^	76.82 ± 0.86 ^a,B^	76.00 ± 0.41 ^a,A,B^
	RBS	78.27 ± 0.93 ^b,B^	76.48 ± 2.14 ^a,A^	77.49 ± 0.45 ^b,A,B^	77.92 ± 0.89 ^a,B^	76.41 ± 0.53 ^a,A^
a*	CBS	11.30 ± 0.32 ^b,D^	10.97 ± 0.53 ^a,C,D^	10.82 ± 0.18 ^b,B,C^	10.41 ± 0.26 ^b,A,B^	10.25 ± 0.32 ^a,A^
	RBS	10.65 ± 0.27 ^a,C^	10.32 ± 0.73 ^a,B,C^	10.06 ± 0.13 ^a,A,B^	9.84 ± 0.26 ^a,A^	10.02 ± 0.25 ^a,A,B^
b*	CBS	10.08 ± 0.15 ^a,C^	9.90 ± 0.47 ^a,B,C^	9.46 ± 0.15 ^a,A^	9.69 ± 0.13 ^a,A,B^	10.01 ± 0.30 ^a,B,C^
	RBS	10.98 ± 0.23 ^b,C^	10.56 ± 0.35 ^b,A^	10.36 ± 0.19 ^b,A^	10.89 ± 0.23 ^b,B,C^	10.61 ± 0.18 ^a,A,B^
pH	CBS	6.35 ± 0.03 ^a,A^	6.37 ± 0.04 ^a,A,B^	6.39 ± 0.03 ^a,A,B^	6.41 ± 0.02 ^a,B^	6.49 ± 0.04 ^a,C^
	RBS	6.61 ± 0.02 ^b,A^	6.64 ± 0.03 ^b,A,B^	6.67 ± 0.03 ^b,B^	6.69 ± 0.04 ^b,B^	6.81 ± 0.03 ^b,C^

* Different lowercase letters indicate significant differences between sausage formulations (*p* < 0.05) for the same storage period, while different uppercase letters are indicative of significant differences between sampling times for the same sausage formulation (*p* < 0.05); CBS—control Bologna sausages; RBS—reformulated Bologna sausages.

**Table 5:** Cooking loss and moisture retention of control and reformulated Bologna sausages *.

	CBS	RBS
Cooking loss (%)	0.68 ± 0.06 ^a^	1.53 ± 0.28 ^b^
Moisture retention (%)	67.84 ± 0.63 ^b^	65.44 ± 0.49 ^a^

* Different superscript letters in the same raw indicate significant differences between sausage formulations (*p* < 0.05). CBS—control Bologna sausages; RBS—reformulated Bologna sausages.

**Table 6:** Textural parameters of control and reformulated Bologna sausages *.

Parameters	CBS	RBS
Hardness (N)	42.83 ± 5.34 ^a^	52.56 ± 7.6 ^b^
Springiness (%)	0.68 ± 0.10 ^a^	0.81 ± 0.02 ^b^
Resilience (adm)	1.66 ± 0.13 ^a^	1.93 ± 1.15 ^b^
Cohesiveness (adm)	1.00 ± 0.01 ^a^	1.00 ± 0.04 ^a^
Gumminess (N)	29.19 ± 1.85 ^a^	42.46 ± 6.20 ^b^
Shear force (N)	21.55 ± 1.22 ^a^	27.51 ± 2.17 ^b^

* Different superscript letters in the same raw indicate significant differences between sausage formulations (*p* < 0.05). CBS—control Bologna sausages; RBS—reformulated Bologna sausages.

**Table 7:** Sensory attributes and overall acceptability scores of control and reformulated Bologna sausages *.

Parameters	CBS	RBS
Color	8.58 ± 0.51 ^a^	8.17 ± 0.72 ^a^
Taste	8.17 ± 0.72 ^a^	8.08 ± 0.67 ^a^
Flavor	8.33 ± 0.78 ^a^	8.50 ± 0.80 ^a^
Texture	8.33 ± 0.78 ^a^	8.08 ± 0.79 ^a^
Overall acceptability	8.33 ± 0.65 ^a^	8.25 ± 0.75 ^a^

* Different superscript letters in the same raw indicate significant differences between sausage formulations (*p* < 0.05). CBS—control Bologna sausages; RBS—reformulated Bologna sausages.

## Data Availability

Data is contained within the article.

## Supplementary Materials

The following supporting information can be downloaded at: https://www.mdpi.com/article/10.3390/foods12183455/s1, Figure S1: Appearance of the emulsion gel; Figure S2: Appearance of control and reformulated Bologna sausages immediately after processing; CBS—control Bologna sausages; RBS—reformulated Bologna sausages.
