# Supplementary material for: Reformulation of Bologna Sausage by Total Pork Backfat Replacement with an Emulsion Gel Based on Olive, Walnut, and Chia Oils, and Stabilized with Chitosan
Source: Foods. 2023 Sep 16;12(18):3455. doi: 10.3390/foods12183455 (PMC10529321; doi:10.3390/foods12183455)
Supplement: Supplementary file 1 [file foods-12-03455-s001.zip › foods-2589986-supplementary.pdf]

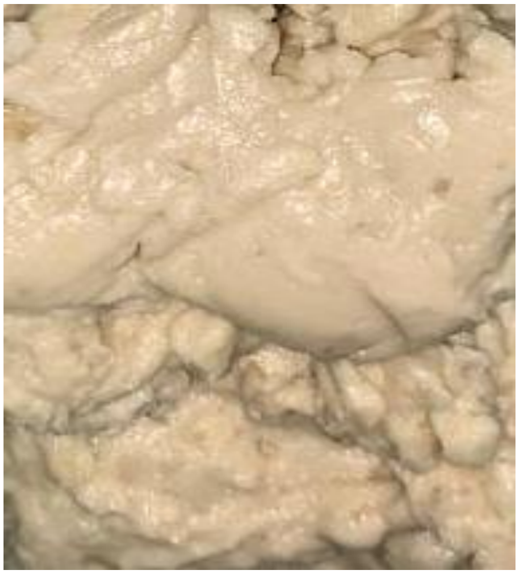

Figure S1. Appearance of the emulsion gel.

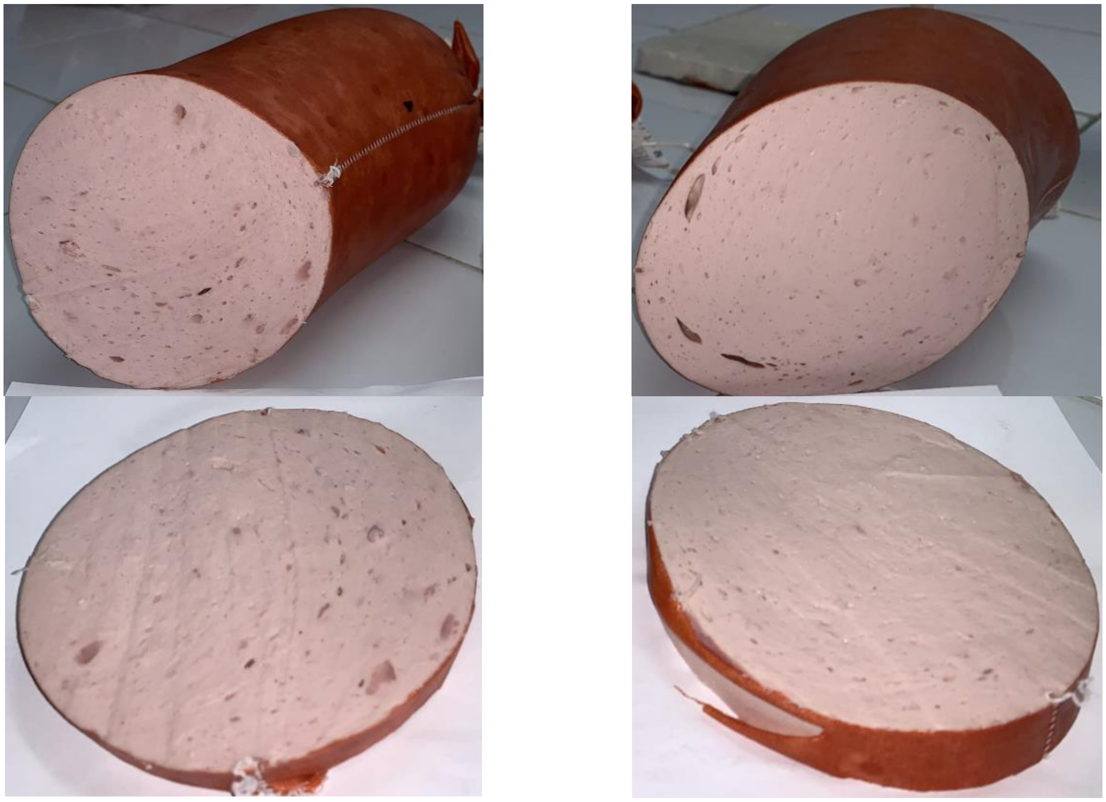

CBS RBS

Figure S2. Appearance of control and reformulated Bologna sausages immediately after processing; CBS—control Bologna sausages; RBS—reformulated Bologna sausages.
